# A positive association between RDW and coronary heart disease in the rheumatoid arthritis population: A cross-sectional study from NHANES

**DOI:** 10.1097/MD.0000000000037315

**Published:** 2024-03-08

**Authors:** Mei Qi Zhang, Wen Tao Tan, Wei Dong Li, Xuan Yang Shen, Yuan Shen, Xiao Lu Jiang, Hong Fu Wen

**Affiliations:** a Department of Emergency, Affiliated Hospital of North Sichuan Medical College, Nanchong, Sichuan, P.R. China.

**Keywords:** CHD, RA, RDW and atherosclerosis

## Abstract

Previous research has indicated that higher red blood cell distribution width (RDW) increases the risk of coronary heart disease. However, no studies have established a link between RDW and coronary heart disease in the rheumatoid arthritis population. This research aims to explore the association between RDW and coronary heart disease among individuals with rheumatoid arthritis. We selected demographic data, laboratory data, lifestyle, and medical history from the National Health and Nutrition Examination Survey (NHANES), specifically including age, gender, poverty, RDW, race, BMI, diabetes, education, coronary heart disease, hypertension, cholesterol, smoking, and drinking. RDW and coronary heart disease were found to have a positive association in the rheumatoid arthritis population (OR = 1.145, 95%CI: 1.036–1.266, *P* = .0098), even after adjusting for factors such as age, gender, race, education level, smoking, and drinking. Subgroup analysis showed a stronger positive association, particularly in individuals aged 55–66 years, males, and the Hispanic White population with diabetes or hypercholesterolemia. There is a significant correlation between RDW and coronary heart disease among individuals with rheumatoid arthritis.

## 1. Introduction

Coronary heart disease (CHD) results from atherosclerotic plaque formation in coronary arteries, causing lumen stenosis and leading to cardiomyocyte ischemia. Plaque rupture exposes subendothelial collagen to blood, triggering platelet aggregation, coronary thrombosis, vascular occlusion, and cardiomyocyte necrosis. Recently, changing lifestyles have contributed to an increasing incidence of CHD, now recognized as one of the most common and serious diseases threatening human health worldwide and a leading cause of death.^[1,2]^

RDW, a routine blood test parameter calculated as the standard deviation of red cell volume divided by the mean cell volume, expressed as a percentage (RDW%), reflects the variability in red blood cell size.^[3]^ Beyond hematological disorders, RDW is associated with various acute and chronic cardiovascular diseases, including coronary heart disease, heart failure, and atrial fibrillation.^[4]^ RDW is implicated in chronic inflammation and is closely linked with patient mortality and the progression of cardiovascular events, serving as an independent predictor of outcomes and mortality in these events.^[5–8]^

Rheumatoid arthritis (RA), a chronic systemic inflammatory disease, affects approximately 0.5% to 1.0% of the global population. Characterized by prolonged duration, involvement of multiple tissues, and a high disability rate, RA significantly impacts patient health.^[9]^ Patients with RA experience disordered immune function and systemic vascular infiltration by inflammatory factors, resulting in an incidence of vascular events 1.5 to 2.0 times higher than the general population.^[10]^

However, limited research exists on the relationship between increased RDW and the severity of coronary artery lesions in CAD patients with RA, including those with complications from RA. It remains unclear whether RDW is an independent risk factor for CHD in RA patients. This study aims to analyze the correlation of RDW with RA to investigate RDW diagnostic value in CHD patients with RA.

## 2. Methods

### 2.1. Population

This study utilized data from the National Health and Nutrition Examination Survey (NHANES) collected between 2011 and 2020.^[11]^

**Figure 1.**
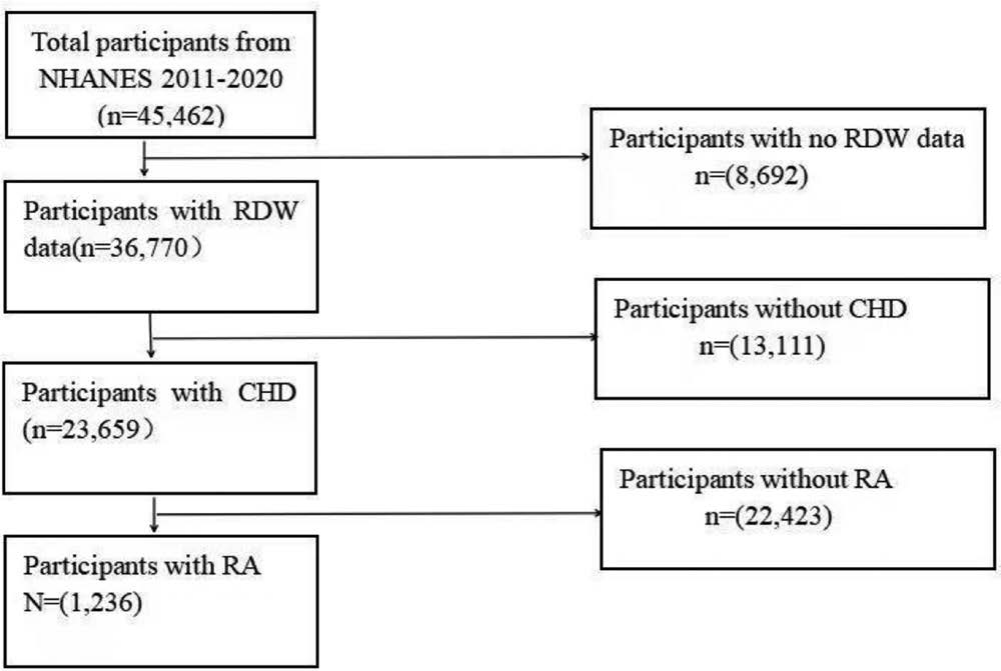
Flow chart of subjects selection from the NHANES 2011 to 2020. NHANES = the National Health and Nutrition Examination Survey.

A total of 45,462 participants were involved in the NHANES survey over all 4 cycles. In our study, RDW values were missing for 8692 participants, CHD values for 13,111, and RA values for 22,423. Ultimately, our sample comprised 1236 individuals (Fig. 1).

### 2.2. Variables

RDW examination was conducted using a questionnaire. RDW tertiles were classified as low, middle, and high. RA diagnosis was based on reports from a physician or other healthcare professional.

Considered in this study were demographic data, laboratory data, lifestyle, and medical history.^[12]^ Demographic data included age, gender, race, poverty, and education level.

Lifestyle factors covered smoking, drinking, and moderate recreational activities. “Drinking” refers to regular alcohol consumption, and “smoking” to individuals who have smoked at least 100 cigarettes in their lifetime.^[13]^

The study also included a history of diabetes, hypertension, and hypercholesterolemia.^[14]^

### 2.3. Statistical analysis

Data analysis was performed using a weighted approach. Both univariate and multivariate logistic regression were employed to assess the relationship between RDW and CHD. A general additive model and subgroup analysis were also utilized. R version 4.0.4 was used for all statistical analyses. A *P* value of .05 was deemed statistically significant.

## 3. Results

The total number of participants in this study was 1236 (Table 1). The mean age of the examined population was 61.107 years. Our findings indicate that factors such as age, gender, BMI, race, diabetes, hypertension, and hypercholesterolemia are strongly associated with the occurrence of CHD. However, poverty, education level, smoking, physical activity, and alcohol consumption were not significantly associated with CHD risk.

**Table 1:** Characteristics of participants in the study.

RDW tertile	Low	Middle	Hight	*P*
N	402	408	425	
Age (yr)	57.858 ± 13.323	61.027 ± 13.636	62.915 ± 12.729	<.001
Poverty	2.131 ± 1.500	2.094 ± 1.450	2.051 ± 1.423	.734
BMI (kg/m²)	30.024 ± 6.652	30.593 ± 6.749	33.144 ± 9.152	<.001
Gender				.001
Male	183 (45.522%)	191 (46.814%)	151 (35.529%)	
Female	219 (54.478%)	217 (53.186%)	274 (64.471%)	
Race				<.001
Mexican American	64 (15.920%)	63 (15.441%)	34 (8.000%)	
Hispanic White	150 (37.313%)	154 (37.745%)	130 (30.588%)	
Hispanic Black	90 (22.388%)	101 (24.755%)	207 (48.706%)	
Other Race	98 (24.378%)	90 (22.059%)	54 (12.706%)	
Education				.229
Less than high school	62 (15.423%)	60 (14.706%)	47 (11.059%)	
High school	150 (37.313%)	169 (41.422%)	183 (43.059%)	
University and above	190 (47.264%)	179 (43.873%)	195 (45.882%)	
Hypertension				<.001
Yes	212 (52.736%)	256 (62.745%)	305 (71.765%)	
No	190 (47.264%)	152 (37.255%)	120 (28.235%)	
Smoking				.904
Yes	205 (50.995%)	213 (52.206%)	223 (52.471%)	
No	197 (49.005%)	195 (47.794%)	202 (47.529%)	
Drinking				.952
Yes	78 (19.403%)	80 (19.608%)	84 (19.765%)	
No	249 (61.940%)	243 (59.559%)	258 (60.706%)	
Other	75 (18.657%)	85 (20.833%)	83 (19.529%)	
Diabetes				.039
Yes	113 (28.109%)	123 (30.147%)	153 (36.000%)	
No	289 (71.891%)	285 (69.853%)	272 (64.000%)	
Coronary heart disease				.024
No	381 (94.776%)	370 (90.686%)	382 (89.882%)	
Yes	21 (5.224%)	38 (9.314%)	43 (10.118%)	
Hypercholesterolemia				.039
Yes	211 (52.488%)	224 (54.902%)	197 (46.353%)	
No	191 (47.512%)	184 (45.098%)	228 (53.647%)	

All continuous variables presented in Table 1 were mean ± standard deviation and categorical variables are presented as n (%). BMI = body mass index, RDW = red blood cell distribution.

### 3.1. Univariate analysis

Table 2 presents the results of the univariate analysis. These results suggest that age, gender, race, RDW, BMI, diabetes, hypertension, and hypercholesterolemia are significantly related to the high incidence of CHD in the RA population (*P* < .05).

**Table 2:** Univariate logistic regression analysis of all variables with coronary heart disease among the rheumatoid arthritis population.

Variables	OR	Lower 95%CI	Upper 95%CI	*P*
Age (yr)	1.077	1.047	1.108	<.0001
Poverty	1.205	1.001	1.450	.0529
BMI (kg/m²)	0.988	0.960	1.016	.4009
RDW	1.145	1.036	1.266	.0098
Gender				
Male	Reference			
Female	0.235	0.130	0.422	<.0001
Race				
Mexican American	Reference			
Hispanic White	1.730	0.557	5.374	.3462
Hispanic Black	0.819	0.292	2.297	.7055
Other Race	0.544	0.187	1.581	.2671
Education				
Less than high school	Reference			
High school	0.662	0.240	1.829	.4296
University and above	1.417	0.585	3.434	.4432
Activity				
Yes	Reference			
No	1.131	0.717	1.785	.5984
Smoking				
Yes	Reference			
No	0.578	0.324	1.030	.0671
Drinking				
Yes	Reference			
No	0.809	0.450	1.455	.4817
Diabetes				
Yes	Reference			
No	0.427	0.259	0.702	.0013
Hypertension				
Yes	Reference			
No	0.341	0.172	0.675	.0029
Hypercholesterolemia				
Yes	Reference			
No	0.412	0.215	0.791	.0096

95%CI = 95% confidence interval, BMI = body mass index, CHD = coronary heart disease, OR = odds ratio, RDW = red blood cell distribution.

### 3.2. The association between RDW and CHD

Multivariate logistic regression was employed to assess the association between RDW and CHD in RA patients (Table 3). Our findings revealed that in Model 3, RDW was positively associated with CHD after adjusting for all confounding factors (OR = 1.187, 95%CI: 1.065–1.322, *P* = .0029). Additionally, we categorized RDW into 3 groups to further investigate its association with CHD. According to the fully adjusted model, an increase in RDW correlates with a higher incidence of CHD.

**Table 3:** Multiple logistic regression analysis of the relationship between red blood cell distribution width and coronary heart disease among the rheumatoid arthritis population.

Exposure	Model 1			Model 2			Model 3		
	OR	95%CI	*P*	OR	95%CI	*P*	OR	95%CI	*P*
RDW	1.145	(1.036, 1.266)	.0098	1.168	(1.060, 1.287)	.0026	1.187	(1.065, 1.322)	.0029
P for trend	<0.001			<0.001			<0.001		

Model 1: we did not adjust any covariants; Model 2: we adjusted for age, gender, and race; Model 3: we adjusted all covariants in Table 1.

### 3.3. The linear relationship between RDW and CHD

**Figure 2.**
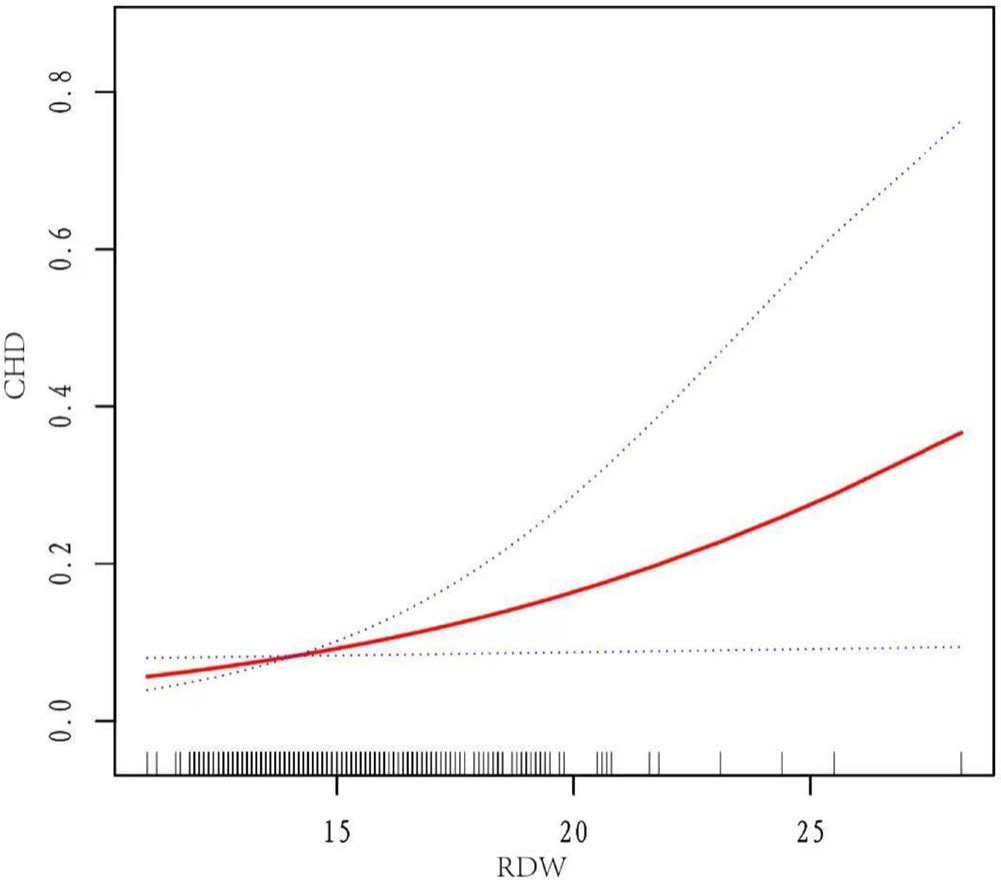
The linear relationship between red blood cell distribution width and coronary heart disease.

A general additive model was used to illustrate the relationship between RDW and CHD in the RA population. Figure 2 shows an increased risk of CHD with higher RDW levels in RA patients.

### 3.4. Subgroup analysis

This section explores the relationship between RDW and CHD across various age groups, genders, races, and the presence of diabetes, hypertension, or hypercholesterolemia (Table 4).

**Table 4:** Subgroup analysis of the relationship between red blood cell distribution width and coronary heart disease among the rheumatoid arthritis population.

	OR	95%CI	*P*
Age (yr)			
20–55	1.129	(0.770, 1.654)	.5342
56–66	1.342	(1.066, 1.690)	.0124
67–80	1.046	(0.853, 1.283)	.6644
Gender			
Male	1.221	(1.034, 1.442)	.0184
Female	1.081	(0.887, 1.319)	.4401
Race			
Mexican American	1.305	(0.895, 1.905)	.1669
Hispanic White	1.255	(1.021, 1.543)	.0313
Hispanic Black	1.122	(0.903, 1.394)	.2986
Other Race	0.849	(0.517, 1.396)	.5197
BMI (kg/m²)			
<=24.9	1.096	(0.875, 1.371)	.4250
>24.9, <=30	1.200	(0.859, 1.675)	.2845
>30	1.130	(0.948, 1.348)	.1730
Diabetes			
Yes	1.035	(0.854, 1.256)	.7241
No	1.204	(1.026, 1.414)	.0231
Hypertension			
Yes	1.123	(0.978, 1.289)	.1003
No	1.226	(0.929, 1.619)	.1500
Hypercholesterolemia			
Yes	1.061	(0.886, 1.269)	.5207
No	1.262	(1.049, 1.519)	.0138

We adjusted all covariants in Table 1.

95%CI = 95% confidence interval, BMI = body mass index, CHD = coronary heart disease, OR = odds ratio, RDW = red blood cell distribution.

We found that in the RA population aged 56 to 66 years, a higher RDW was more strongly associated with CHD (OR = 1.342, 95%CI: 1.066–1.690, *P* = .0124), based on age group stratification. In gender and race subgroup analyses, significant associations were observed between RDW and CHD in males (OR = 1.221, 95%CI: 1.034–1.442, *P* = .084), and Hispanic Whites (OR = 1.255, 95%CI: 1.021–1.543, *P* = .0313).

## 4. Discussion

There is a significant association between RDW and the incidence of CHD in the RA population, indicating that higher RDW levels correlate with an increased risk of CHD. Subgroup analysis revealed a more pronounced association in males, Hispanic Whites, and individuals aged 56 to 66.

Rheumatoid arthritis is primarily characterized by synovitis and vasculitis, affecting multiple systems. Studies have highlighted that the cardiovascular system is one of the most frequently affected organs in RA patients, with cardiovascular events being the leading cause of mortality,^[15]^ often linked to atherosclerotic disease. Subclinical coronary atherosclerosis^[16]^ is common in RA patients. However, atherosclerosis is a systemic, chronic process where early detection and intervention are crucial for reducing the incidence and mortality of coronary heart disease in RA patients.

Inflammation serves as a common pathological basis for both RA and coronary atherosclerosis. Inflammatory factors are key pathogenic elements,^[17]^ impacting vascular endothelium, liver, and other tissues. They increase blood viscosity, homocysteine (Hcy) levels, and insulin resistance, thereby promoting coronary atherosclerosis.^[18]^ Consequently, RA presents a heightened risk for CHD. Given RA global prevalence, investigating CHD risk in RA patients is of significant practical importance. This research establishes an association between RDW and CHD in the RA population.

Subgroup analysis identified high-sensitivity groups. A significant positive correlation between RDW and CHD was found in individuals aged 56 to 66. The association was strongest among RA men and Hispanic Whites.

However, this study has limitations. While it demonstrates an association between RDW and CHD, the cross-sectional design precludes definitive conclusions about causality. Additionally, despite including numerous confounding factors, some were not considered in our study.

## 5. Conclusion

A positive correlation exists between RDW and CHD in the RA population, with a stronger association observed in individuals aged 56–66, males, and the Hispanic White population, particularly those with diabetes or hypercholesterolemia.

## Author contributions

**Conceptualization:** Meiqi Zhang.

**Data curation:** Meiqi Zhang, Wentan Tan, Yuan Shen.

**Formal analysis:** Xiaolu Jiang.

**Funding acquisition:** Weidong Li.

**Methodology:** Meiqi Zhang.

**Resources:** Xuanyang Shen.

**Software:** Meiqi Zhang, Xuanyang Shen, Hongfu Wen.

**Supervision:** Weidong Li.

**Writing – original draft:** Meiqi Zhang.

**Writing – review & editing:** Meiqi Zhang.
